# A novel tool for monitoring endogenous alpha-synuclein transcription by NanoLuciferase tag insertion at the 3′end using CRISPR-Cas9 genome editing technique

**DOI:** 10.1038/srep45883

**Published:** 2017-04-04

**Authors:** Sambuddha Basu, Levi Adams, Subhrangshu Guhathakurta, Yoon-Seong Kim

**Affiliations:** 1 Division of Neurosciences, Burnett School of Biomedical Sciences, College of Medicine, University of Central Florida, 6900 Lake Nona Blvd, Orlando, FL 32827, USA; 2 Kyunghee University Medical College, Seoul, South Korea

## Abstract

α-synuclein (α-SYN) is a major pathologic contributor to Parkinson’s disease (PD). Multiplication of α-SYN encoding gene (*SNCA*) is correlated with early onset of the disease underlining the significance of its transcriptional regulation. Thus, monitoring endogenous transcription of *SNCA* is of utmost importance to understand PD pathology. We developed a stable cell line expressing α-SYN endogenously tagged with NanoLuc luciferase reporter using CRISPR/Cas9-mediated genome editing. This allows efficient measurement of transcriptional activity of α-SYN in its native epigenetic landscape which is not achievable using exogenous transfection-based luciferase reporter assays. The NanoLuc activity faithfully monitored the transcriptional regulation of *SNCA* following treatment with different drugs known to regulate α-SYN expression; while exogenous promoter-reporter assays failed to reproduce the similar outcomes. To our knowledge, this is the first report showing endogenous monitoring of α-SYN transcription, thus making it an efficient drug screening tool that can be used for therapeutic intervention in PD.

α-synuclein (α-SYN) is a key protein involved in the progression and pathogenesis of Parkinson’s disease (PD)^1^. Familial PD studies have revealed that multiple copies of the gene encoding α-SYN (*SNCA*) cause severe early onset PD, highlighting the importance of its tight transcriptional control^2^,^3^,^4^,^5^. However, little is known about the transcriptional dysregulation of *SNCA*. Recently, Gründemann *et al*. confirmed significant increase in *SNCA* mRNA levels in individual dopamine neuron from idiopathic PD brains using laser capture microdissection, implying a significant transcriptional de-regulation in pathologic conditions^6^. Moreover, recent progress in research on epigenetic influences on *SNCA* transcription revealed that hypomethylation of *SNCA* regulatory region play a significant role towards its higher expression in idiopathic PD^7^,^8^,^9^.

Advances in genome mapping and the completion of ENCODE project (Encyclopedia of DNA Elements) highlighted the importance of epigenetic architecture governing transcriptional regulation of a gene^10^,^11^. In light of these discoveries, complete understanding of *SNCA* expression in pathologic conditions may require a molecular tool/system that can detect changes in transcription and also account for changes brought about by endogenous epigenetic modulation of the gene. Currently, the most widely used tool for understanding transcriptional activity of a gene is by using luciferase reporter fused to the promotor of a gene of interest^12^. However, the plasmid-based exogenous reporter systems largely ignore the comprehensive aspect of gene expression regulation by complex interaction between different epigenetic factors, transcription factors and various *cis* elements by artificially limiting investigation on a putative promoter region. To overcome this limitation of exogenous reporter system, we developed a novel tool where a reporter construct is tagged at the 3′end of *SNCA* endogenously, allowing us to monitor transcriptional activity of the gene keeping its epigenetic architecture unperturbed. The NanoLuc luciferase reporter used in this study is 150-fold brighter and significantly smaller in size than *firefly* or *Renilla* luciferase, thus making it an ideal tag for even low expressing genes^13^,^14^. Recent breakthrough in genome editing techniques like CRISPR/Cas9 (clustered regularly interspaced short palindromic repeats) have made specific genome editing simple and scalable^15^. Tagging *SNCA* endogenously with the NanoLuc using CRISPR/Cas9 method allows sensitive and real-time measurement of changes in transcriptional activity under various conditions of stimuli. This strategy can help to shed light on the transcriptional regulation of *SNCA*, and may serve as a very strong tool for screening of drugs to limit the progression of PD.

## Results

### Generation of a stable cell line endogenously tagged with functional NanoLuc luciferase reporter at the 3′end of *SNCA*

**Figure 1:**
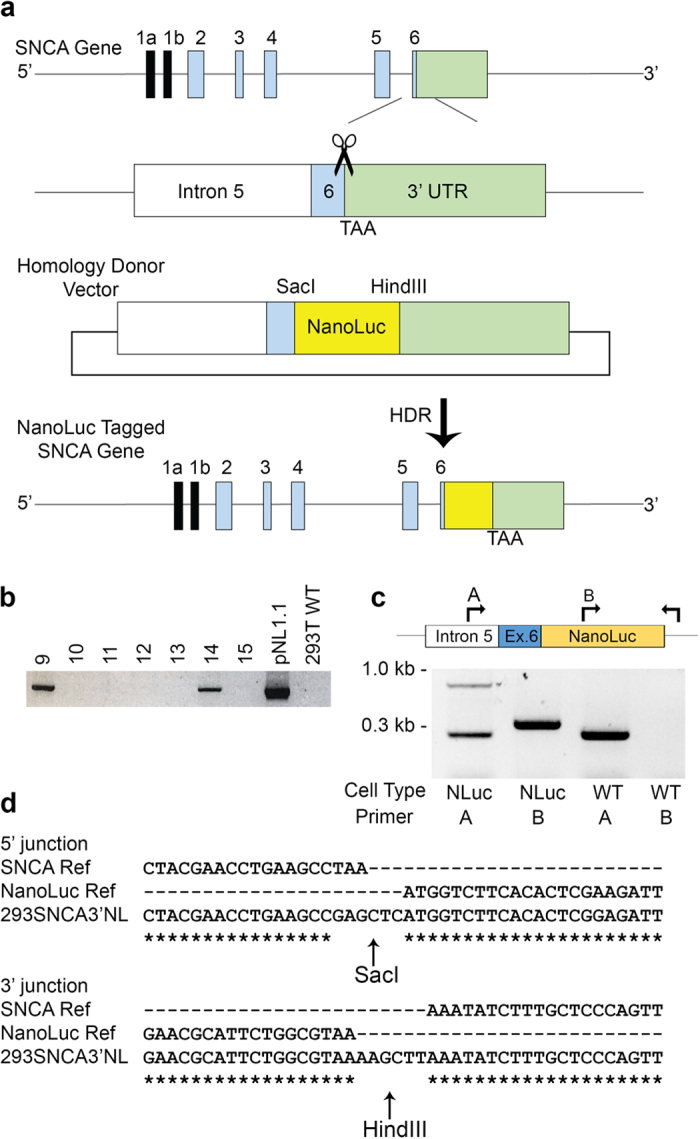
Development of 293T-*SNCA*-3′NL cells. (**a**) Schematic representation of cloning strategy. *SNCA* gene map showing exons (1a and 1b non-coding, 2–6 coding) and the 3′UTR. Transfection of sgRNA targeting the 3′end of exon 6 induces a DSB near the stop codon (TAA). Donor vector design contains 5′ homology arm of 790 bp encompassing part of intron 5 and exon 6 upstream from the stop codon and the NanoLuc-3′ homology arm of 800 bp downstream of the stop codon containing part of the 3′UTR. Co-transfection of donor vector with the CRISPR/Cas9 construct precisely incorporated the NanoLuc right before the stop codon by HDR of the *SNCA* gene. (**b**) Following puromycin selection and single cell dilution, genomic DNA from all surviving isogenic colonies were screened for the NanoLuc insert with pNL1.1 NanoLuc vector and HEK293T LVX cells as controls. From 15 colonies recovered, two were positive for the NanoLuc insertion. (**c**) Gene specific PCR with primers in the intron 5 (A) and the 3′UTR of *SNCA* showed colony 9 had a heterozygous insertion in 293T-SNCA-3′NL cells (Lane 1); PCR with forward primer on the NanoLuc (B or NanoLuc Internal Forward Primer) and the same 3′UTR reverse primer (cDNA sequencing Reverse Primer) showed comparable amplification of the NanoLuc tagged allele (Lane 2); PCR of the wild-type α-SYN and NanoLuc from the HEK293T LVX as controls (Insertion Confirmation Forward Primer and cDNA sequencing Reverse Primer) (Lanes 3 and 4) (**d**) Excerpt of Sanger sequencing results showing insertion of the NanoLuc sequence with restriction sites precisely before the stop codon of *SNCA* and with correct continuation of the 3′UTR after the NanoLuc sequence.

To introduce the NanoLuc reporter at the 3′end of the *SNCA*, a double-strand DNA break (DSB) was introduced on the reverse strand with the –NGG protospacer adjacent motif sequence (PAM) directly abutting the stop codon located in the exon 6. This was achieved in human embryonic kidney cell line (HEK293T) by transient transfection with the pSpCas9 (BB)-2A-Puro vector^16^. Along with the CRISPR/Cas9 construct, a donor vector containing the NanoLuc sequence was co-transfected to take advantage of the cell’s homology-directed repair (HDR)^17^. This donor construct contained two flanking homology domains each of about 800 base pairs, corresponding to the upstream and downstream of the DSB target site (Fig. 1a). The NanoLuc sequence was cloned between these two domains to precisely introduce this reporter construct right before the stop codon of *SNCA*. Potential positive clones were identified by PCR amplification of the genomic DNA using the NanoLuc forward and reverse primers (Table 1) (Fig. 1b; Supplementary Fig. 1). To confirm NanoLuc insertion at the target location in the *SNCA* gene, a second PCR using “Insertion Confirmation Primers” (Table 1) was performed and later sequenced (Supplementary Fig. 1). The wild-type (WT) allele generated a 280 base pair (bp) PCR amplicon while the NanoLuc-tagged allele generated a 805 bp band, indicating a heterozygous insertion of the reporter construct (Fig. 1c). To overcome the PCR amplification bias towards the shorter allele, a separate amplification for NanoLuc-tagged allele was performed using a forward primer (NanoLuc Internal Forward Primer) on the NanoLuc insert in combination with the same reverse primer (cDNA sequencing Reverse Primer) on the 3′UTR, generated a comparable amplification of 356 bp product for NanoLuc insert (Fig. 1c, lane 2). The PCR using NanoLuc internal forward primer failed to amplify any band in wild type HEK293T cells (Fig. 1c, lane 4). A second potential positive clone was found to have an incomplete insertion of the NanoLuc reporter tag (colony 14, Fig. 1b), and thus not used any further. To confirm the expression of NanoLuc-tagged *SNCA* allele tagged with the NanoLuc in the cell line, hereafter referred to as 293T-*SNCA*-3′NL, RT-PCR was conducted using primers encompassing the entire coding region of the gene and the 3′ UTR. The amplicon was then sequence verified to confirm the presence of NanoLuc insertion (Fig. 1d, Supplementary Fig. 2).

**Figure 2:**
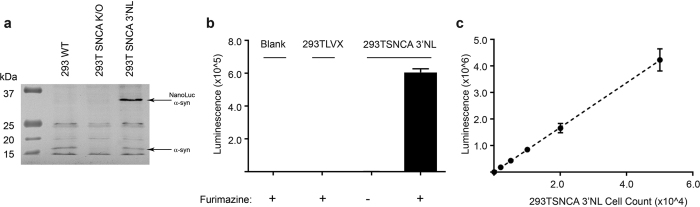
Functional expression of the NanoLuc luciferase. (**a**) Lysates from HEK293T LVX, HEK293T *SNCA* K/O, and 293T-*SNCA*-3′NL cells were Western blotted with anti-α-SYN antibody. As expected, a wild-type band appeared in HEK293T LVX and 293T-*SNCA*-3′NL cells, but in the 293T-*SNCA*-3′NL cells an additional band was identified at approximately 34 kDa corresponding to α-SYN fused with the NanoLuc that was absent in non-NanoLuc tagged cells. (**b**) Luminescence activity of HEK293T LVX cells compared with 293-*SNCA*-3′NL cells. Only 293T-*SNCA*-3′NL cells generate significant NanoLuc activity in the presence of furimazine as the substrate. (**c**) Luminescence of 293T-*SNCA*-3′NL cells follows a linear trend with increasing cell numbers (R^2^ = 0.95). All the experiments have been performed in triplicate.

Following confirmation of the NanoLuc insertion in the *SNCA* genomic region and the presence of mature mRNA, we sought to confirm protein expression and functional activity of the NanoLuc. Western blot analysis of cell lysates with a polyclonal anti α-SYN antibody confirmed the presence of both wild type α-SYN (~15 KDa) and a NanoLuc-tagged protein (~34 KDa) matching α-SYN fused with the NanoLuc (19.1 KDa) (Fig. 2a, Supplementary Fig. 3). We performed luciferase activity assay on 293T-*SNCA*-3′NL cells by measuring luminescence after addition of substrate furimazine. 293T-*SNCA*-3′NL cells produced a considerably high signal distinguishable from cells without the NanoLuc incorporation or 293T-*SNCA*-3′NL cells without furimazine (Fig. 2b). Titration of cell counts from 2,500 to 50,000 produced a linear increase in luminescence activity (R^2^ = 0.95), indicating that the luminescence of the *SNCA*-tagged NanoLuc reporter is internally consistent (Fig. 2c).

Taken together, these results show that *SNCA* was successfully tagged with the NanoLuc construct at the 3′end, and that expression of the NanoLuc-tagged allele leads to generation of a fusion protein.

### α-SYN-NanoLuc luciferase activity reflects *SNCA* transcriptional regulation

**Figure 3:**
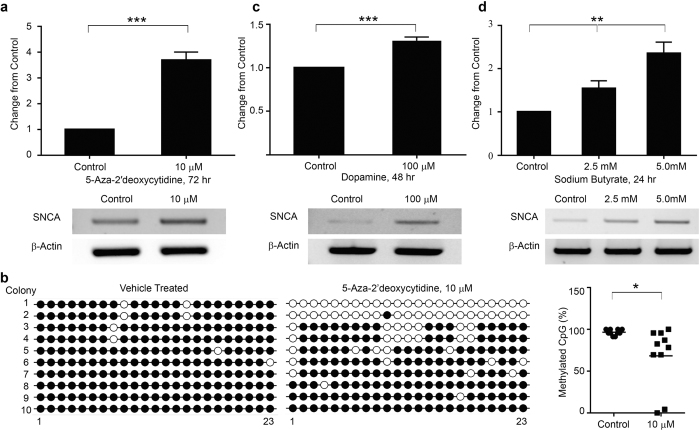
293T-*SNCA*-3′NL cells having the NanoLuc integration can be used to model deregulated *SNCA* as seen in sporadic PD. (**a**) 293T-*SNCA*-3′NL cells treated with 10 μM 5-AzadC for 72 hours which induced a significant increase in the NanoLuc activity as compared to the control. The NanoLuc activity was corroborated by increase in α-SYN transcript as shown in the RT-PCR. (**b**) Methylation status of 23 CpG sites on the *SNCA* intron1 was determined by bisulfite sequencing. Amplified PCR products were cloned into pGEM-T Easy vector and 10 clones were sequenced. (Left) Comparison of vehicle and 5-AzadC treatment (10 μM, 72 hours) showing unmethylated (open circles) and methylated (closed circles) cytosines for all 10 clones (y-axis) at each of the 23 CpGs in intron1 (x-axis). (Right) Scatter plot showing overall decrease in methylation by 31.7% compared to the control. (**c**) Similarly, 293T-*SNCA*-3′NL cells treated with dopamine at 100 μM concentration for 48 hours, increased NanoLuc activity significantly. Increase in the NanoLuc activity was confirmed by RT-PCR after dopamine treatment. (**d**) Following HDAC inhibitor (sodium butyrate) treatment at concentrations of 2.5 mM and 5.0 mM for 24 hours, the 293T-*SNCA*-3′NL cells showed a significant dose dependent increase in the NanoLuc activity. This dose-dependent increase in the NanoLuc activity was also confirmed by RT-PCR following same treatment paradigm. β-actin amplification was used as an internal control for all the PCRs. Error bars show the mean from three technical repeats. p values are given for t-test (5-AzadC, dopamine), one-way ANOVA (Sodium butyrate) where *represents p < 0.05, **represents p < 0.01, ***represents p < 0.0001.

To validate whether this system would be able to monitor changes in endogenous *SNCA* transcription, 293T-*SNCA*-3′NL cells were treated with known epigenetic modulators like DNA methyltransferase 1(DNMT1) inhibitor (5-AzadC), histone deacetylase (HDAC) inhibitors (sodium butyrate) and also dopamine which may have a toxic effect beyond a certain threshold concentration 18. *SNCA* harbors CpG islands at the regulatory regions encompassing the promoter and intron1^8^,^9^. The CpG island in the intron1of *SNCA* in HEK293T remains completely methylated which upon demethylation can increase gene expression^9^. We treated the 293T-*SNCA*-3′NL cells with 5-AzadC for 72 hours to allow more than one round of cell division. A significant increase in the NanoLuc activity was observed in cells treated with 5-AzadC as compared to vehicle treated ones (3.68 times increase; p ≤ 0.0001) (Fig. 3a). *SNCA* transcript level from sister cultures correlated well with the observed increase in the NanoLuc activity (Fig. 3a). Changes in methylation of the *SNCA*-intron1 CpG island were detected using bisulfite sequencing as done by Jowaed *et al*.^8^. Ten clones from each sample were analyzed and a significant reduction in mean intron 1 methylation by 31.7% (p < 0.05) was observed (Fig. 3b). The reduction of cytosine methylation in the intron1 positively correlated with increase in *SNCA* transcript, as we saw with the increased NanoLuc activity.

It has already been reported that dopamine at 100 μM concentration can enhance *SNCA* transcription in HEK293T cells without inducing subsequent toxicity^9^,^18^. Likewise, we treated 293T-*SNCA*-3′NL cells with 100 μM dopamine for 48 hours and a significant 1.31 times increase in the NanoLuc activity was observed as compared to the controls (p < 0.0001) (Fig. 3c). Again this increase in the NanoLuc luciferase activity complied with an increasing trend in α-SYN/Nanoluc mRNA and protein expression after dopamine treatment as seen by RT-PCR and western blot analyses (Fig. 3c, Supplementary Fig. 4). Hyper-acetylation of histone is expected to unwind underlying DNA, which in turn favors transcription^19^,^20^. To test whether HDAC inhibition in 293T-*SNCA*-3′NL cells would faithfully monitor transcription in response to histone hyper-acetylation, cells were treated with sodium butyrate (class I and IIa inhibitor of HDAC) at concentrations 2.5 mM and 5.0 mM for 24 hours^19^. This treatment paradigm significantly increased the NanoLuc activity by 1.5 and 2.35 times respectively as compared to the controls (p < 0.001) (Fig. 3d). α-SYN/NanoLuc transcript levels also corroborated well with the activity measurement and showed a dose-dependent increase (Fig. 3d).

### Exogenous promoter reporter assays failed to reproduce transcriptional activation of *SNCA* as seen in endogenous conditions

**Figure 4:**
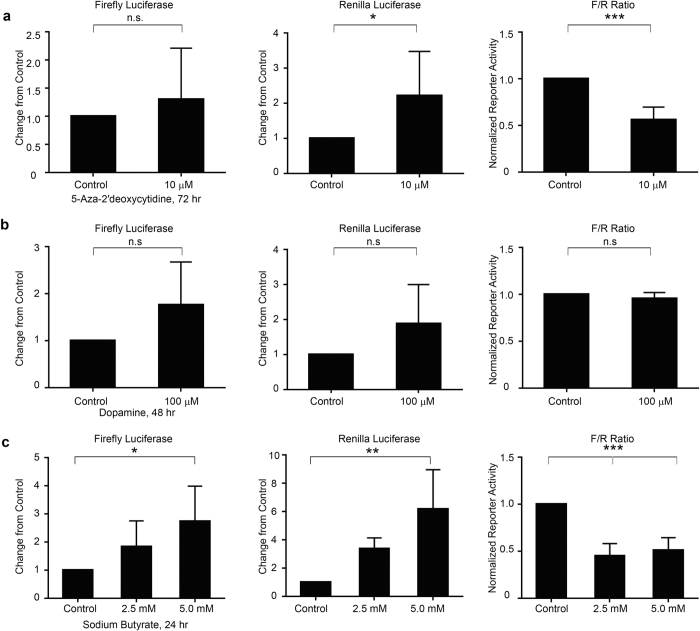
Exogenous overexpression of *SNCA* driven *firefly* and *Renilla* luciferase failed to replicate endogenous *SNCA* behavior after similar paradigm of drug treatment. (**a**) HEK293T LVX cells were co-transfected with *SNCA*-pGL3 (*firefly* luciferase) and CMV-pRL (*Renilla* luciferase) constructs. Twenty-four hours after transfection, cells were subjected to 10 μM 5-AzadC treatment for 72 hours. No significant change in *firefly* luciferase activity was seen, however the *Renilla* luciferase activity showed a significant increase. Thus overall normalized luciferase assay showed a significant decrease in the activity (F/R) after treatment with 5-AzadC. (**b**) Similarly, HEK293T LVX cells were also co-transfected with *SNCA*-pGL3 and CMV-pRL plasmids, treated dopamine at concentration of 100 μM for 48 hours. No significant change was observed either in *firefly* luciferase activity or *Renilla* luciferase activity. Thereby, the normalized luciferase activity (F/R) also did not show any significant change after treatment with dopamine. (**c**) Treatment with sodium butyrate at concentrations of 2.5 mM and 5.0 mM respectively for 24 hours, gave comparable results (like 5-AzadC) in normalized luciferase activity (F/R) which showed a significant decrease. This decrease in the normalized luciferase was attributed to the significant increase in *firefly* luciferase activity and *Renilla* luciferase activity. Error bars show the mean from three technical repeats. p values given for t-test (5-AzadC, dopamine), one-way ANOVA (Sodium butyrate) for three independent experiments where *represents p < 0.05, **represents p < 0.01, ***represents p < 0.0001.

*Firefly* luciferase-based promoter assay is considered a gold standard for assessing promoter activity of a target gene, which in turn reflects the transcriptional activity of the gene^21^. We compared the reporter activity of *SNCA* transcription between transient transfection-based luciferase system and the endogenous NanoLuc system that we designed. HEK293T cells were co-transfected with pGL3 basic plasmid containing *SNCA* promoter-intron1 region cloned upstream of luciferase coding sequence and CMV-*Renilla* (transfection control). Twenty-four hours later, transfected cells were treated exactly with the same modulators of *SNCA* expression as described in the previous result. We observed a significant decrease in normalized reporter activity upon 5-AzadC treatment post 72 hrs (p < 0.0001) (Fig. 4a) contrary to the increased NanoLuc activity and transcript expression (Fig. 3a). The CMV promoter-driven *Renilla* luciferase activity which was used as an internal control significantly varied upon 5-AzadC treatment, while the *SNCA* promoter-intron1 driven *firefly* luciferase activity remained largely unaffected, thereby leading to reduction in the normalized reporter activity (*firefly/Renilla*) (Fig. 4a). Next, to compare the effect of dopamine on the exogenous luciferase activity driven by *SNCA* promoter-intron1 and the endogenous *SNCA* behavior, the transfected cells were treated with 100 μM dopamine for 48 hrs, exactly following paradigm followed for the 293T-*SNCA*-3′NL cells. Interestingly, we did not observe any significant change in the normalized luciferase activity (p = 0.22) (Fig. 4b). In this treatment, no significant change was observed for either *firefly* luciferase (p = 0.15) or *Renilla* luciferase activity (p = 0.16) (Fig. 4b), although an increasing trend could be seen for both. This data again failed to demonstrate the endogenous state of regulation upon dopamine treatment (Fig. 3c).

We also investigated the effect of HDAC inhibition (for 24 hrs) on the *SNCA* promoter-intron1 driven luciferase activity. Similar to 5-AzadC treatment, normalized *firefly* activity showed a significant decrease from control (p < 0.0001) (Fig. 4c). This time we saw a significant increase in the *firefly* luciferase activity (p < 0.05), along with a significant increase in the *Renilla* activity (p < 0.001) thereby causing an artefactual reduction in *SNCA* promoter activity after normalization (p < 0.0001). This observation is again opposite to what we found with the NanoLuc-based endogenous system after addition of HDAC inhibitor (Fig. 3d). Together, these observations showed that the exogenous luciferase-based reporter system largely failed to demonstrate original state of endogenous transcriptional activity of *SNCA*.

## Discussion

In the present study, we have successfully incorporated the NanoLuc reporter construct at the 3′end of *SNCA* in HEK293T cells using CRISPR/Cas9 technology and monitored changes in expression induced by two epigenetic modulators and dopamine which are known to deregulate the gene’s expression. The newly emerging endogenous reporter system represents a significant paradigm shift in the study of gene regulation and may provide new and exciting opportunities for both basic and translational research. Such strategies allow to insert a reporter directly into the targeted genome, enabling us to investigate endogenous gene regulation while keeping the epigenetic structure intact.

This particular feature is extremely relevant in studying *SNCA* expression, as this gene has been shown to get extensively regulated by its epigenetic structure^7^,^8^,^9^. Moreover, α-SYN is a molecule whose level of expression is directly correlated with the severity of the PD pathogenesis, thus making this tool very helpful for developing potential therapeutic options^2^,^3^,^4^,^6^. Recent studies have demonstrated that epigenetic regulation by 5-AzadC increases *SNCA* expression in SK-SN-SH and SH-SY5Y cells^8^,^22^. This cytidine analogue passively demethylates by irreversibly trapping DNA methyltransferase I (DNMTI), which positively correlates with higher expression of various genes^23^. *SNCA* harbors CpG islands at the regulatory regions encompassing the promoter and intron1^8^,^9^. It has been shown that hypomethylation of the intron1 CpG island but not the promoter CpG island is strongly associated with PD^8^. So, we sought to check the methylation status of the 23 CpG sites^8^ in the *SNCA*-intron1 by bisulfite sequencing. As expected, a decrease in methylation of intron1 CpG correlated with an increase in transcript levels as measured by the NanoLuc activity. Conventionally, to study the effect of methylation on the promoter’s transcriptional efficiency, that promoter-reporter construct is fully methylated *in vitro* and the reporter activity is then compared with the unmethylated one^8^,^24^. Consistent with our observation for endogenous α-SYN with nanoluc activity, Jowaed *et al*. showed that *in*-*vitro* methylation of regulatory promoter-reporter construct of α-SYN reduces transcription of α-SYN. Studying gene expression this way could lead to different outcome than actual transcriptional state of the target gene as the regulatory region of that gene might exist as partially methylated condition endogenously, which cannot be replicated using the exogenous system. Moreover, comprehensive regulation of the target gene promoter with inputs coming from other local epigenetic modifications such as histone post translational modifications (PTMs)^25^ and *trans* factors which are usually present endogenously, might not be able to regulate the exogenously introduced promoter of the same gene.

During genetic typing, we saw that the intensity of the NanoLuc-tagged allele after PCR is relatively weaker when compared to the wild-type PCR product for both the genomic DNA and cDNA (Fig. 1c and Supplementary Fig. 2a). This may be a result of PCR bias towards the amplification of the shorter allele over the longer ones involving reaction in a single tube^26^. This problem of preferential amplification was overcome by using separate primer set to amplify only the NanoLuc-tagged allele (not encompassing the wild-type allele, Fig. 1c and Supplementary Fig. 4a,b) which could amplify the NanoLuc-tagged allele very efficiently and comparably to the wild-type allele (Fig. 1c and Supplementary Fig. 4b). In addition to using separate set of primers, we used equal number of cycles (30 cycles) to ensure that the PCR products are not saturated, which is indicated by the increase in expression after dopamine treatment. However, the western blot shown in the Fig. 2a indicated that the NanoLuc-tagged α-SYN protein (band shown at ~34 KDa) is higher in intensity than the wild type protein (band at ~15 KDa) when probed with α-SYN specific antibody. This unequal distribution of protein bands between wild type (low) and NanoLuc-tagged allele (high) may indicate that either that the Nanoluc luciferase has been targeted to α-SYN in more than one allele of the locus, or it is also possible that fusion of a highly stable NanoLuc luciferase protein with α-SYN may affect the stability of the target protein positively. HEK293T cells are not typically diploid, and are instead complex hypo-triploid in nature, containing less than three times the number of chromosomes of a normal diploid human cell^27^. Our results show that the nanoluc reporter construct is precisely targeted to the end of *SNCA* and faithfully reports the transcriptional changes of α-SYN.

The advantage of using an endogenous reporter system to study regulation of transcription by epigenetic environment of the gene was highlighted by the data obtained from conventional reporter assays after treatment with different drugs such as 5-AzadC, dopamine and sodium butyrate.

As 5-AzadC inhibits DNA methylation in dividing cells therefore we previously observed that the drug reduced endogenous *SNCA*-intron1 methylation and increased transcription or nanoluc activity in our endogenous reporter system. However, we did not observe such increase in exogenous *SNCA*-promoter/intron1 *firefly* reporter activity upon drug treatment, may be due to lack of DNA methylation in the exogenous plasmid (Fig. 4a first panel). Surprisingly, we observed a significant increase in *Renilla* luciferase activity upon 5-AzadC treatment (Fig. 4a second panel) and a significant decrease in normalized reporter activity (Fig. 4a third panel). However, the observed decrease in reporter activity can be attributed to CMV-*Renilla* luciferase activity which, although equally transfected, significantly varied upon 5-AzadC treatment. The mechanism of this apparent variation in *Renilla* luciferase activity is yet unknown. However, it is known that *Renilla* luciferase activity can vary significantly upon treatment paradigm depending on the promoter driving its activity. This could lead to erroneous interpretation of the observed data after normalization to *Renilla* as is a standard protocol for these type of assays^28^. Therefore it was suggested to use different or multiple endogenous controls in case of employing exogenous reporter systems^28^.

It was also shown that dopamine can enhance α-SYN transcription in HEK293 cells^9^. Similarly, we also observed a significant increase in the NanoLuc activity upon dopamine treatment to 293T-*SNCA*-3′NL cells (Fig. 3b) unlike exogenous promoter-reporter assay (Fig. 4b), which further fortifies the reliability of our endogenous tagging system. This apparent discrepancy between the outcome coming from endogenous and exogenous systems may be attributed to either lack of appropriate crosstalk between *cis/trans* elements in exogenous reporter system or due to lack of methylation structure in the *SNCA* promoter-intron1 construct in the plasmid-based luciferase system. Since, the aim of our study was to design a tool that can faithfully monitor changes in endogenous α-SYN transcription at physiological level, the question that we seek to address was whether this NanoLuc-tagged α-SYN reflected accurately the change in transcription of wild-type α-SYN (although present in higher copy) after treatment with dopamine. The comparable increasing trend of mRNA and protein of both the wild-type and the tagged α-SYN after treatment with dopamine (Supplementary Fig. 4b,c) indicated that the engineered cell line could efficiently replicate transcriptional changes of endogenous wild-type α-SYN.

Next we investigated how HDAC inhibitor, sodium butyrate can regulate transcription of *SNCA*. As the dynamics between histone acetylation or chromatin relaxation and histone deacetylation or chromatin condensation play an important role in regulation of gene transcription^29^,^30^, it can be envisaged that the application of HDAC inhibitor would result in hyper-acetylated condition in the gene promoter, which in turn might favor induction in transcription. As expected, we observed a significant increase in the NanoLuc activity in response to sodium butyrate (p < 0.001) (Fig. 3d). However, a significant opposite observation was noticed using exogenous reporter system (Fig. 4c). This prominent decrease in the normalized reporter activity can again be attributed to the significant increase of *Renilla* luciferase activity following sodium butyrate treatment. Interestingly, it has been shown that this HDAC inhibitor can change the overall chromatin structure of the cell and functions through the butyrate response elements on the gene regulatory regions that encompass Sp1/Sp3 binding sites^31^. Since, *SNCA* regulatory region contains multiple Sp1/Sp3 binding sites, it is reasonable to assume that recruitment of acetylated Sp1/Sp3 can enhance *SNCA* expression. Analysis of CMV promoter sequence revealed that it harbors around 5 cognate sequences for Sp1/Sp3 binding, suggesting a possible mechanism for sodium butyrate mediated significant increase in the *Renilla* luciferase activity. Since, *Renilla* luciferase plasmid is usually used as endogenous control for this kind of conventional luciferase assay, it is very important to interpret the reporter assay results with caution using this type of transfection controls^28^.

Transcriptional upregulation of *SNCA* has long been a concern in PD pathogenesis. Most intellectual effort has been focused on aggregation behavior, resulting in a lacuna of information on transcriptional regulation of this gene despite its immense importance. In this study we developed a novel screening tool that can efficiently monitor *SNCA* transcript levels under different treatment conditions known to up-regulate transcription. This tool can provide a new diagnostic platform for drug development and testing of compounds believed to regulate *SNCA* in the cell in an inexpensive and precise way. As the epigenetic environment for this gene regulation is kept unchanged, the effects of treatments would more closely mimic the state seen in the cells than has previously been available. It is also worth mentioning that the stability and sensitivity of this nanoluc luciferase reporter makes it suitable to monitor very low expressing genes which is unlikely to be achievable by any other conventional reporter systems. At this juncture it is also important to mention that our study highlights the importance of using endogenous reporter system over exogenous reporter system particularly for studying comprehensive epigenetic regulation of gene transcription. Specifically this *SNCA*-NL reporter system is very useful to study the effects of drugs or agents that are known to modulate gene’s chromatin structure or any other epigenetic environment. Therefore previous studies which used exogenous *firefly/Renilla*-based reporter system to demonstrate the important *cis* elements of the *SNCA* promoter or novel transcription factors and pathways for the gene are still useful^32^,^33^,^34^,^35^.

## Methods

### Cell Culture

HEK293T LVX cells were maintained in DMEM (Dulbecco’s Modified Eagle Medium) supplemented with heat-inactivated 10% fetal bovine serum and 1% Penicillin/Streptomycin (Gibco, 10000 U/mL). Cells were maintained at 37 °C in humidified incubators with 5% CO_2_ and passaged following trypsinaization with 0.25% Trypsin/0.53 mM EDTA (ThermoFisher Scientific).

### Designing *SNCA* specific short guide RNA (sgRNA)

The vector for cloning of the sgRNA pSpCas9 (BB)-2A-Puro (PX459) was a gift from Feng Zhang Lab (Addgene plasmid # 48139)^16^. The sgRNA targeting the stop codon area of the *SNCA* gene was ligated into the pSpCas9(BB)-2A-Puro vector as previously described with minor modifications^16^ (Table 1). Briefly, sgRNA oligos corresponding to 20 base pairs immediately upstream of –AGG PAM sequence located on the reverse strand at the *SNCA* stop codon were ligated into pSpCas9(BB)-2A-Puro vector using Fast-Digest BbsI (ThermoFisher Scientific) and T7 ligase (New England Biolabs) by cycling between 37 °C and 21 °C 10 times for 6 minutes. Ligation reaction was treated with Plasmid-Safe ATP-Dependent DNase (Epicentre) and transformed into CaCl_2_ competent cells DH5α *E. coli* by heat shock (42°, 50 sec). Plasmid was purified using Gene-Jet plasmid Miniprep Kit (ThermoFisher Scientific) according to manufacturer’s protocol and successful insertion of the sgRNA was confirmed by sequencing.

### Cloning of NanoLuc-homology donor vector

Primers used for amplification, addition of restriction sites and insert confirmation are listed in Table 1. Approximately 800 bp-length homology domains were amplified from HEK293T genomic DNA using Q5-Polymerase (New England Biolabs). Upstream homology arm (Chromosome 4, 89,727,231–89,728,021, reverse strand GRCh38:CM000666) was modified to include a 5′ NotI restriction site and a 3′ SacI restriction site (Fig. 1). Downstream homology arm (Chromosome 4, 89,726,457–89,727,235, reverse strand GRCh38:CM000666) was modified to include a 5′ HindIII cut site and 3′ NotI/AatII cut site. The NanoLuc luciferase sequence was amplified from pNL1.1 vector (a gift from Promega Corporation). To allow for sequential plasmid ligation, NanoLuc coding sequence was modified to include terminal 5′ SacI and 3′ HindIII/AatII restriction enzyme sites. Upstream homology arm and the NanoLuc were ligated together and cloned into NotI and AatII digested pGEM -T Easy vector (Promega Corporation; cat no. A137A) and transformed into competent DH5α cells. Plasmid was purified using Gene-Jet Plasmid Miniprep Kit according to manufacturer’s protocol (ThermoFisher Scientific). Downstream homology arm was then cloned into AatII-digested vector. Completed homology sequence was digested with NotI to release sequence from pGEM -T Easy vector and delete AatII sequence, then ligated into NotI digested pAAV-IRES-hrGFP backbone (2,846 bp) and transformed into chemically competent DH5α cells. Presence of insert was confirmed by sequencing.

### Generation of HEK-293T cell line stably expressing *SNCA*-NanoLuc (293T-*SNCA*-3′NL)

Vector constructs were transfected into HEK293T LVX (CloneTech) cells using X-Fect Polymer (CloneTech) according to manufacturer’s protocol. Briefly, HEK293T LVX cells were seeded in a 6-well plate with 1 × 10^6^ and allowed to grow to 80% confluency. X-Fect Polymer was mixed with 1.25 μg pSpCas9 (BB)-2A-Puro with *SNCA* sgRNA and 1.25 μg NanoLuc-homology arm donor vector and incubated with cells for 4 hours, followed by a change with fresh media. After 48 hours, cells were subjected to 5 μg/mL puromycin (ThermoFisher Scientific, cat no. AC227420100) selection for 48 hours. Surviving puromycin resistant cells were diluted to a single cell level and plated in 96 well plate then allowed to propagate for approximately two weeks. 15 pure colonies were recovered and grown to 50% confluency before passaging them to 1:2 in a 24 well plate.

### Confirmation of stable integration of the NanoLuc reporter at the 3′end of *SNCA*

Genomic DNA of the puromycin resistant colonies was extracted by 16 hours incubation in lysis buffer (10 mM Tris-HCl pH 7.6, 0.5 mM EDTA, 0.67% SDS, 132 μg/ml Proteinase-K) at 55 °C, followed by precipitation in 100% ethanol with 150 mM NaCl. Presence of the NanoLuc insert was confirmed by PCR using NanoLuc forward and reverse cloning primers and identity of insert was confirmed by gene specific confirmation primers in intron 5′ and the 3′ UTR of *SNCA* using PCR master mix (GenDepot, P0311-200). The details of the “Insert Confirmation primers” are listed in Table 1. Individual bands were excised and sequenced to confirm the sequence to ensure introduction of any mutations and location of insert in the genomic DNA and cDNA (Macrogen USA).

### Western blotting

For Western blotting, protein was extracted using RIPA (Radio Immuno Precipitation buffer) buffer (PBS, 1% NP-40, 0.5% Sodium deoxycholate, 0.1% PMSF, 100 ng/ml protease inhibitor, dH_2_O) and 40 μg protein were ran on 10% denaturing SDS gel and transferred to PVDF membrane (Millipore cat no. IPFL00010). Following the transfer process, the membrane was fixed in 0.4% PFA in PBS as suggested by Lee *et al*.^36^. The membrane was then blocked with Odyssey Blocking Buffer (LI-COR cat no. 927-50000) mixed 1:1 with TBS. Specific protein bands were detected using rabbit anti-α-SYN antibodies (Santa Cruz Biotechnology, cat no. SC-7011R, 1:1000 dilution) overnight and followed by goat anti-rabbit secondary antibodies for fluorescent detection at 680 nm (LI-COR, cat no. 926-68020). Western blotting for the experiments related to 48 hour dopamine treatment, proteins were extracted as before and transferred to PVDF membrane. Membrane was blocked with 5% milk in TBS-T. Specific protein bands were detected using mouse anti-α-SYN antibodies (BD Transduction Laboratories, cat no. 610786) at 1:250 dilution, followed by goat anti-mouse HRP secondary antibodies for chemiluminescent detection (Jackson Immuno Research laboratories Inc. cat no. 115-035-146).

### Assay for the NanoLuc luciferase activity

To measure the NanoLuc luciferase activity, 200,000 cells were seeded per well of a 24 well plate. After 24 hours, appropriate treatments were done and incubated for designated periods of time in duplicate. Cells were detached by trypsinization and two wells were combined in a 1.5 mL microcentrifuge tube and pelleted at 2,000 × g for 3 minutes. Cell pellets were resuspended in 500 μL colorless DMEM and counted using an automated cell counter (BIORAD, USA) three times and then luciferase activity was monitored in technical triplicate using the Nano-Glo Luciferase Assay System according to manufacturer’s protocol with minor modifications (Promega corporation, cat no. N1110). Briefly, 5,000 cells in 30 μL colorless DMEM were assayed using 30 μL of assay buffer mixed 1:100 with NanoGlo substrate. Plates were incubated for 5 minutes and then luminescence was recorded in triplicate on a multi-plate reader (EnVision, PerkinElmer). This experiment was performed three times independently.

### *SNCA* promoter-reporter assay

To generate the *SNCA* promoter luciferase construct, *SNCA* promoter-intron1 region (−2,200 to +118 bp; with respect to ATG) was amplified from HEK293T genomic DNA and cloned into XhoI/HindIII sites of promoter less pGl3 basic vector (Promega, USA, cat no. E1751). The presence of insert was validated by sequencing. For the promoter-reporter assays, 200,000 HEK293T LVX cells were co-transfected with *SNCA*-pGL3 and CMV-pRL (as a transfection control) vectors in a 24 well plate format. Each well were co-transfected with 500 ng of *SNCA*-pGL3 and 10 ng of CMV-pRL plasmids using X-Fect polymer as described above and incubated 24 hours before proceeding to any chemical/drug treatments. Following the drug treatments for appropriate time HEK293T LVX cells were collected for lysis. Briefly, cell pellet was lysed in 100 μL of Lysis Buffer (25 mM Tris-Phosphate Buffer pH 8.0, 4 mM EGTA, 2 mM DTT, 20% Glycerol & 1% Triton X-100) for 10 minutes at room temperature with occasional shaking, then centrifuged at 16,000 × g for 5 minutes and the lysate was collected in a separate tube. 10 μL of this lysate was assayed with 140 μL of luciferase assay buffer (25 mM Tris-Phosphate Buffer pH 8.0, 4 mM EGTA, 20 mM MgSO_4_, 1 mM DTT, and 2 mM ATP). *Firefly* luciferase activity (0.75 mM luciferin, 10 mM DTT) and *Renilla* luciferase activity (1.5 μM Coelenterazine, 100 mM NaCl, 25 mM Tris pH 7.5) were measured in triplicate using a multi-plate reader (EnVision, PerkinElmer). Relative luciferase activity was measured by normalizing the *firefly* luciferase activity to *Renilla* luciferase activity (F/R).

### Cell treatment paradigm

All the treatments were performed in duplicate wells. Compounds used in the study are as follows: Sodium butyrate (Alfa Aeser; cat no. A11079), (5-AzadC) (Sigma, cat no. A3656) and Dopamine hydrochloride (Sigma, cat no. H8502). Sodium butyrate stock was prepared in distilled water at a concentration of 500 mM and cells were treated with sodium butyrate for 24 hours at 2.5 mM and 5.0 mM. 5-AzadC stock of 5 mM was prepared in 50% acetic acid and immediately aliquoted and frozen. Cells were treated with 10 μM 5-AzadC for 72 hours, refreshed every 8–12 hours^8^. Dopamine stock was prepared in distilled water at a concentration of 10 mM. Cells were treated with 100 μM dopamine for 48 hours, refreshed every 24 hours^9^. Control wells were treated with respective vehicles for indicated times and no visible cellular toxicity was noticed under aforementioned treatment paradigms.

### Bisulfite sequencing

Genomic DNA from 293T-*SNCA*-3′NL cells following 5-AzadC treatment was extracted as mentioned above. Approximately, 500 ng high quality DNA (260/280 > 1.8) was used for bisulfite conversion using EZ DNA Methylation-Direct Kit (Zymo Research cat no. D5020) then used as template for PCR amplification of *SNCA* intron1 region using specific primers designed for bisulfite modified DNA as described in article by Jowaed *et al*.^8^. EpiMark Hot Start Taq DNA Polymerase (NEB Inc; cat No. M0490S) was used for PCR and the amplicon was cloned into pGEM -T Easy vector (Promega Corporation). 10 positive colonies were selected for sequencing analysis using T7 universal primer. The sequenced products were analyzed for degree of methylation using BISMA and QUMA software^37^,^38^.

### Semi quantitative reverse transcriptase PCR (RT-PCR)

RNA was extracted using TRIzol Reagent according to manufacturer protocols (Life Technologies Inc.; cat no. 15596-026). Complementary DNA (cDNA) was generated by conversion of 1 μg total RNA using amfiRivert cDNA Synthesis Platinum Master Mix according to manufacturer’s protocol (GenDEPOT; cat no. R5600-50). The cDNA was diluted 1:1 with nuclease free water before PCR. To check the expression of *SNCA*, amplification was done using primers against the NanoLuc sequence and normalized by the amplification of an endogenous control gene, β-actin. The scheme of the differential amplification of NanoLuc-tagged allele and wild type *SNCA* alleles are shown in Supplementary Fig. 4a. Details of all the primers are listed in the Table 1.

### Statistical analysis

Statistical analysis was performed using GraphPad Prism 5 (GraphPad Software Inc.). Data are presented as mean ± SEM. To get statistically meaningful data, all experiments were performed in technical triplicates. The times change in the NanoLuc luciferase activities in treated groups were calculated by normalizing the value with respective control for each experiment. All the experiments were independently repeated three separate times using separate batches of cells and the means of each experimental set were analyzed. In case of exogenous luciferase reporter assays, minimum of four independent repeats were performed. Statistical significance was determined by comparing means of different groups and conditions using unpaired 2-tailed Student’s t test, and one-way ANOVA. Multiple corrections were made using post-hoc Tukey test, Bonferroni test and Scheffe’s test whenever it was required. Significance was assessed at 95% level.

## Additional Information

**How to cite this article:** Basu, S. *et al*. A novel tool for monitoring endogenous alpha-synuclein transcription by NanoLuciferase tag insertion at the 3′end using CRISPR-Cas9 genome editing technique. *Sci. Rep.* **7**, 45883; doi: 10.1038/srep45883 (2017).

**Publisher's note:** Springer Nature remains neutral with regard to jurisdictional claims in published maps and institutional affiliations.

## Supplementary Material

Supplementary Information

## Figures and Tables

**Table 1:** Sequences of the oligo used for the generation and confirmation of 293T-*SNCA*-3′NL cells.

Name of the oligos	Sequence (5′ to 3′)
*SNCA* sgRNA Top Strand	CACCGTGGGAGCAAAGATATTTCTT
*SNCA* sgRNA Bottom Strand	AAACAAGAAATATCTTTGCTCCCAC
Upstream Homology Forward Primer	TATGGCGGCCGCTTAGGAACAAGGAAAAT
Upstream Homology Reverse Primer	AGTGAGCTCGGCTTCAGGTTCGTAGTC
NanoLuc Forward Primer	AAAGAGCTCATGGTCTTCACACTCGAA
NanoLuc Reverse Primer	CATGACGTCAAGCTTTTACGCCAGAATGCGTG
Downstream Homology Forward Primer	AAGCTTAAATATCTTTGCTCCCAGTTTCTTGA
Downstream Homology Reverse Primer	GTTGACGTCGCGGCCGCATACCAAAACA
Insertion Confirmation Forward Primer	CTGCAGAATATTTGCAAAAACATTGATTG
Insertion Confirmation Reverse Primer	TAAAAACTTTGAGAAATGTCATGACTGGG
cDNA sequencing Forward Primer	GGAGTGGCCATTCGACGACAGTG
cDNA sequencing Reverse Primer	TAAAAACTTTGAGAAATGTCATGACTGGG
SNCA Exon 4 Forward Primer	CAAATGTTGGAGGAGCAGTGGTGA
NanoLuc Internal Forward Primer	AAGGTGATCCTGCACTATGGCA
