# Supplementary material for: A novel tool for monitoring endogenous alpha-synuclein transcription by NanoLuciferase tag insertion at the 3′end using CRISPR-Cas9 genome editing technique
Source: Sci Rep. 2017 Apr 4;7:45883. doi: 10.1038/srep45883 (PMC5379209; doi:10.1038/srep45883)

A novel tool for monitoring endogenous alpha-synuclein transcription by NanoLuciferase tag insertion at the 3'end using CRISPR-Cas9 genome editing technique

Sambuddha Basu[1, 3], Levi Adams[1, 3], Subhrangshu Guhathakurta[1*], Yoon-Seong Kim[1,2]

Figure Legends

Supplementary Figure 1 | Sequence alignment of wild type *SNCA* against 293T-*SNCA*-3′NL genomic DNA sequence. Part of the sequence alignment is shown depicting the 3' end of the *SNCA* of 293T-SNCA-3'NL. The wild type (WT) *SNCA* genomic DNA sequence (reference sequence: GRCh38.p7) was aligned with the sequencing data from 293T-SNCA-3′NL to check for the proper incorporation of the NanoLuc construct at the end of the coding sequence of *SNCA*. Sequence 1 is expected *SNCA* genomic DNA, sequence 2 is 293T-SNCA-3′NL sequence. The last exon of *SNCA* is highlighted in blue. The start and stop codons of NanoLuc are highlighted by yellow and red colors respectively. The aligned bases between both the sequences are marked by star (*). The in-frame incorporation of the NanoLuc can be seen in between the two restriction enzyme sites, SacI and HindIII (underlined) in 293T-SNCA-3′NL cells (2). Sequence alignment was done using Clustal Omega program.

Supplementary Figure 2 | 293T-*SNCA*-3′NL cDNA indicates correct insertion of NanoLuc sequence. **a.** PCR of *SNCA* from 293T-*SNCA*-3'NL cDNA using "cDNA sequencing primers" (Table 1) produces two bands, one matching expected wild-type size and one matching expected size for wild-type with NanoLuc insertion (NanoLuc Tagged α-syn). **b.** Part of the sequence alignment is shown depicting the coding region of the gene and incorporation of the Nanoluc sequence in 293T-*SNCA*-3′NL cell line. The WT *SNCA* mRNA sequence (reference sequence:

NM_007308.2, transcript variant 4) was aligned with the sequencing data from 293T-*SNCA*-3′NL to check for the proper incorporation of the NanoLuc construct at the end of the coding sequence of *SNCA*. Sequence (1) is WT *SNCA* mRNA transcript, sequence (2) is 293T-*SNCA*-3′NL sequence. The start and stop codons in the WT *SNCA* mRNA sequence are highlighted by green and red colors respectively. The aligned bases between both the sequences are marked by star (*). The in-frame incorporation of the NanoLuc can be seen in between the two restriction enzyme sites, SacI and HindIII (underlined) in 293T-*SNCA*-3′NL cells (2). The NanoLuc insert is unique to 293T-*SNCA*-3′NL cell line and not found in WT *SNCA* mRNA sequence. The new stop codon after the NanoLuc sequence in 293T-*SNCA*-3′NL cell is marked by yellow. "N" denotes unread base in the sequence. Sequence alignment was done using Clustal Omega program.

Supplementary Figure 3 | Full western blot of 293T-*SNCA*-3'NL cells showing NanoLuc tagged and WT α-synuclein. Full western image from Fig. 2a. α-SYN was detected using polyclonal rabbit antibody (Santa Cruz, SC-7011-R). Wild-type α-SYN is shown at approximately 15 kDa and is present in both wild-type 293T cells and 293T-*SNCA*-3'NL cells, but absent from 293T-*SNCA*-knockout cells. NanoLuc-fused α-SYN is shown at approximately 34 kDa, and only appears in 293T-SNCA-3'NL cells. The unmarked bands present in the blot are non-specific bands that commonly appear with this particular polyclonal antibody.

Supplementary Figure 4 | Comparable increase of endogenous and NanoLuc-tagged *SNCA* levels following dopamine treatment. a. Schematic representation of PCR strategy to avoid

preferential amplification issues from heterozygous allele sizes. For WT amplification, primers used were "*SNCA* Exon 4 Forward" and "Insert Confirmation Reverse" (Table 1) to give an amplification product size of 338 bp. For NanoLuc specific amplification, primer pair used were "NanoLuc Internal Forward" and "Insert confirmation reverse" to get a product size of 356 bp. Elongation time was restricted for NanoLuc specific amplification to prevent competitive wild-type amplification. **b, c.** Qualitative image of RT-PCR and western blot analyses of 293T-*SNCA*-3'NL cDNA and protein under vehicle and dopamine (100 μM) treated condition show comparable increasing trend for both wild-type and NanoLuc tagged α-SYN.

Supplementary Figure 1

```
1       ------------gggagccatttcctatctcattggctgtcagtgctgatgcgtaattga
2       TTAGTGTAAGTGGGGAGCCATTTCCTATCTCATTGGCTGTCAGTGCTGATGCGTAATTGA
                    ************************************************

1       aacttatactaacagtgtgtgctgtctttttgatttttctaatattaggaagggtatcaa
2       AACTTATACTAACAGTGTGTGCTGCCTTTTTGATTTTTCTAATATTAGGAAGGGTATCAA
        ************************ ***********************************

1       gactacgaacctgaagccGAGCTCATGGTCTTCACACTCGAAGATTTCGTTGGGGACTGG
2       GACTACGAACCTGAAGCCGAGCTCATGGTCTTCACACTCGAAGATTTCGTTGGGGACTGG
        ************************************************************

1       CGACAGACAGCCGGCTACAACCTGGACCAAGTCCTTGAACAGGGAGGTGTGTCCAGTTTG
2       CGACAGACAGCCGGCTACAACCTGGACCAAGTCCTTGAACAGGGAGGTGTGTCCAGTTTG
        ************************************************************

1       TTTCAGAATCTCGGGGTGTCCGTAACTCCGATCCAAAGGATTGTCCTGAGCGGTGAAAAT
2       TTTCAGAATCTCGGGGTGTCCGTAACTCCGATCCAAAGGATTGTCCTGAGCGGTGAAAAT
        ************************************************************

1       GGGCTGAAGATCGACATCCATGTCATCATCCCGTATGAAGGTCTGAGCGGCGACCAAATG
2       GGGCTGAAGATCGACATCCATGTCATCATCCCGTATGAAGGTCTGAGCGGCGACCAAATG
        ************************************************************

1       GGCCAGATCGAAAAAATTTTTAAGGTGGTGTACCCTGTGGATGATCATCACTTTAAGGTG
2       GGCCAGATCGAAAAAATTTTTAAGGTGGTGTACCCTGTGGATGATCATCACTTTAAGGTG
        ************************************************************

1       ATCCTGCACTATGGCACACTGGTAATCGACGGGGTTACGCCGAACATGATCGACTATTTC
2       ATCCTGCACTATGGCACACTGGTAATCGACGGGGTTACGCCGAACATGATCGACTATTTC
        ************************************************************

1       GGACGGCCGTATGAAGGCATCGCCGTGTTCGACGGCAAAAAGATCACTGTAACAGGGACC
2       GGACGGCCGTATGAAGGCATCGCCGTGTTCGACGGCAAAAAGATCACTGTAACAGGGACC
        ************************************************************

1       CTGTGGAACGGCAACAAAATTATCGACGAGCGCCTGATCAACCCCGACGGCTCCCTGCTG
2       CTGTGGAACGGCAACAAAATTATCGACGAGCGCCTGATCAACCCCGACGGCTCCCTGCTG
        ************************************************************

1       TTCCGAGTAACCATCAACGGAGTGACCGGCTGGCGGCTGTGCGAACGCATTCTGGCGTAA
2       TTCCGAGTAACCATCAACGGAGTGACCGGCTGGCGGCTGTGCGAACGCATTCTGGCGTAA
        ************************************************************

1       AAGCTTaaatatctttgctcccagtttcttgagatctgctgacagatgttccatcctgta
2       AAGCTTAAATATCTTTGCTCCCAGTTTCTTGAGATCTGCTGACAGATGTTCCATCCTGTA
        ************************************************************

1       caagtgctcagttccaatgtgcccagtcatgacatttctcaaagtttttacagtgtatct
2       CAAGTGCTCAGTTCCAATGTACCCAGTCATGACATTTCTCAAAGTTTTTACAGTGTATCT
        ******************** ***************************************

1       cgaagtcttccatcagcagtgattgaagtatctgtacctgcccccactcagcattt----
2       CGAAGTCTTCCATCAGCAGTGATTGAAGTATCTGTACCTGCCCCCACTCAGCATTTCGGT
        ********************************************************
```

Supplementary Figure 2

a

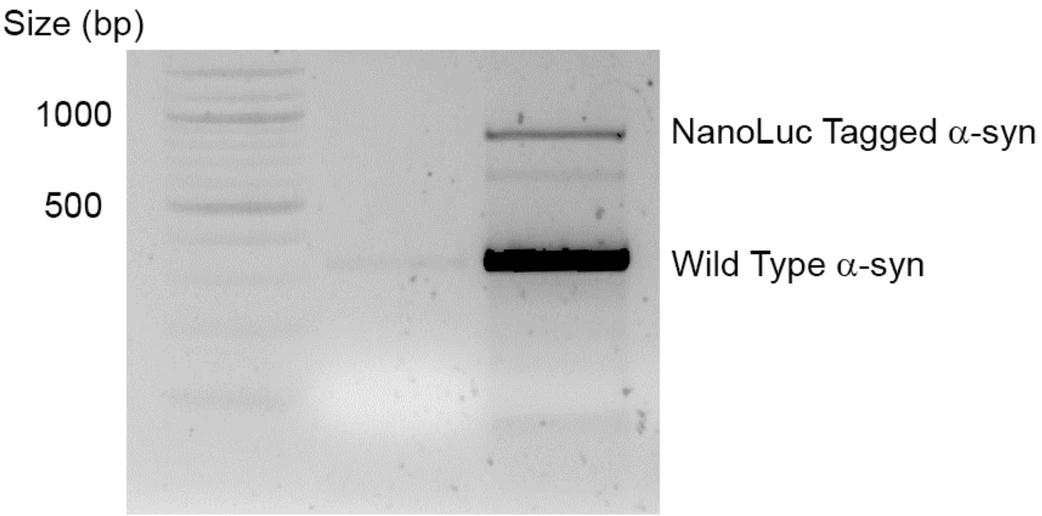

b

```
1       TGGTGTAA-AGGAATTCATTAGCCATGGATGTATTCATGAAAGGACTTTCAAAGGCCAAG
2       TGGTGTAAAGGAATTACATTAGCCATGGATGTATTCATGAAAGGACTTTCAAAGGCCAAG
        ********  * * * ********************************************

1       GAGGGAGTTGTGGCTGCTGCTGAGAAAACCAAACAGGGTGTGGCAGAAGCAGCAGGAAAG
2       GAGGGAGTTGTGGCTGCTGCTGAGAAAACCAAACAGGGTGTGGCAGAAGCAGCAGGAAAG
        ************************************************************

1       ACAAAAGAGGGTGTTCTCTATGTAGGCTCCAAAACCAAGGAGGGAGTGGTGCATGGTGTG
2       ACAAAAGAGGGTGTTCTCTATGTAGGCTCCAAAACCAAGGAGGGAGTGGTGCATGGTGTG
        ************************************************************

1       GCAACAGTGGCTGAGAAGACCAAAGAGCAAGTGACAAATGTTGGAGGAGCAGTGGTGACG
2       GCAACAGTGGCTGAGAAGACCAAAGAGCAAGTGACAAATGTTGGAGGAGCAGTGGTGACG
        ************************************************************

1       GGTGTGACAGCAGTAGCCCAGAAGACAGTGGAGGGAGCAGGGAGCATTGCAGCAGCCACT
2       GGTGTGACAGCAGTAGCCCAGAAGACAGTGGAGGGAGCAGGGAGCATTGCAGCAGCCACT
        ************************************************************

1       GGCTTTGTCAAAAAGGACCAGTTGGGCAAGAATGAAGAAGGAGCCCCACAGGAAGGAATT
2       GGCTTTGTCAAAAAGGACCAGTTGGGCAAGAATGAAGAAGGAGCCCCACAGGAAGGAATT
        ************************************************************

1       CTGGAAGATATGCCTGTGGATCCTGACAATGAGGCTTATGAAATGCCTTCTGAGGAAGGG
2       CTGGAAGATATGCCTGTGGATCCTGACAATGAGGCTTATGAAATGCCTTCTGAGGAAGGG
        ************************************************************

1       TATCAAGACTACGAACCTGAAGCCTAA------------------------------
2       TATCAAGACTACGAACCTGAAGCCGAGCTCATGGTCTTCACACTCGAAGATTTCGTT
```

```
        ************************
1       ------------------------------------------------------------
2       GGGGACTGGCGACAGACAGCCGGCTACAACCTGGACCAAGTCCTTGAACAGGGAGGTGTG

1       ------------------------------------------------------------
2       TCCAGTTTGTTTCAGAATCTCGGGGTGTCCGTAACTCCGATCCAAAGGATTGTCCTGAGC

1       ------------------------------------------------------------
2       GGTGAAAATGGGCTGAAGATCGACATCCATGTCATCATCCCGTATGAAGGTCTGAGCGGC

1       ------------------------------------------------------------
2       GACCAAATGGGCCAGATCGAAAAAATTTTTAAGGTGGTGTACCCTGTGGATGATCATCAC

1       ------------------------------------------------------------
2       TTTAAGGTGATCCTGCACTATGGCACACTGGTAATCGACGGGGTTACGCCGAACATGATC

1       ------------------------------------------------------------
2       GACTATTTCGGACGGCCGTATGAAGGCATCGCCGTGTTCGACGGCAAAAAGATCACTGTA

1       ------------------------------------------------------------
2       ACAGGGACCCTGTGGAACGGCAACAAAATTATCGACGAGCGCCTGATCAACCCCGACGGC

1       ------------------------------------------------------------
2       TCCCTGCTGTTCCGAGTAACCATCAACGGAGTGACCGGCTGGCGGCTGTGCGAACGCATT

1       ---------------GAATATCTTTGCTCCCAGTTTCTTGAGATCTGCTGACAGATGTTC
2       CTGGCGTAAAAGCTTAAATATCTTTGCTCCCAGTTTCTTGAANTCTGCTGACAGATGTTC
                        *************************  *****************

1       CATCCTGTACAAGTGCTCAGTTCCAATGTGCCCAGTCATGACATTTCTCAA
2       CATCCTGTACAAGTGCTCANTTCCAATGTGCCCAGTCATGACATTTCTCAA
        ******************* *******************************
```

Supplementary Figure 3

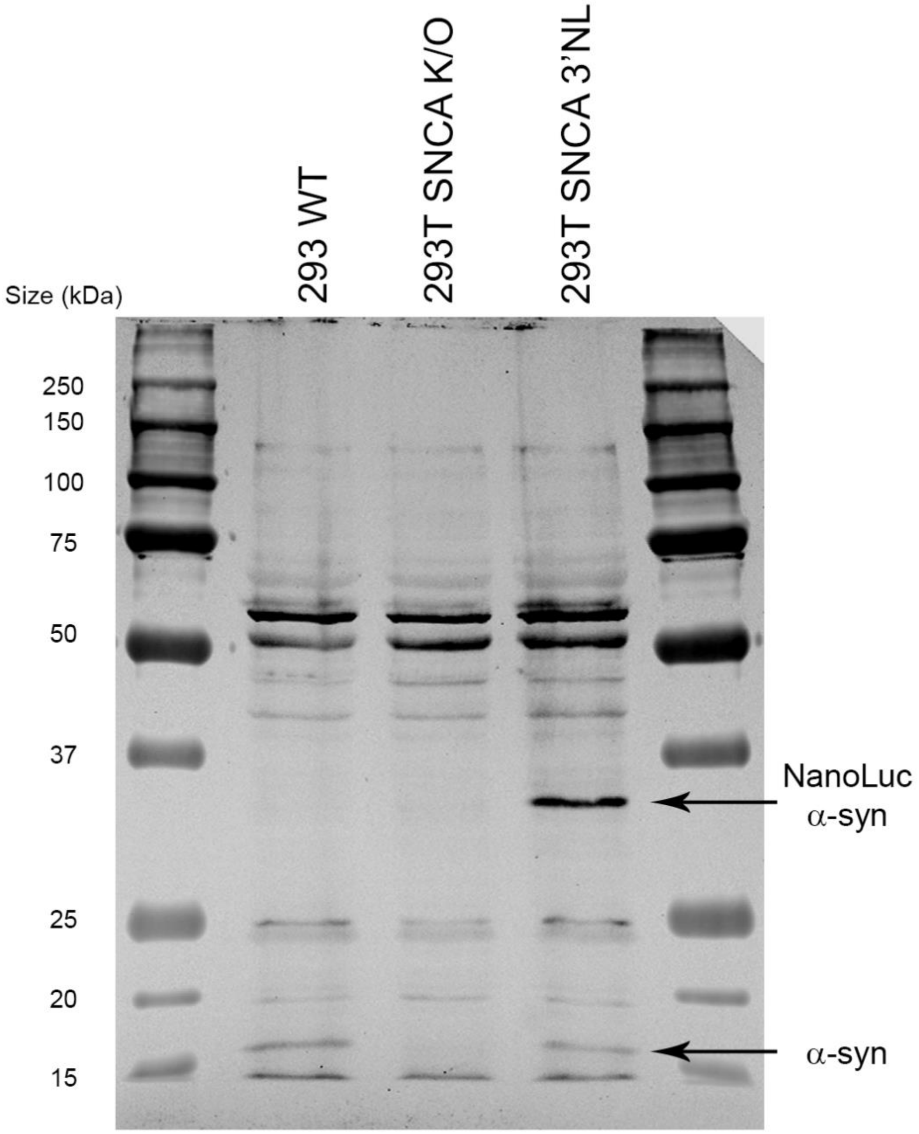

Supplementary Figure 4

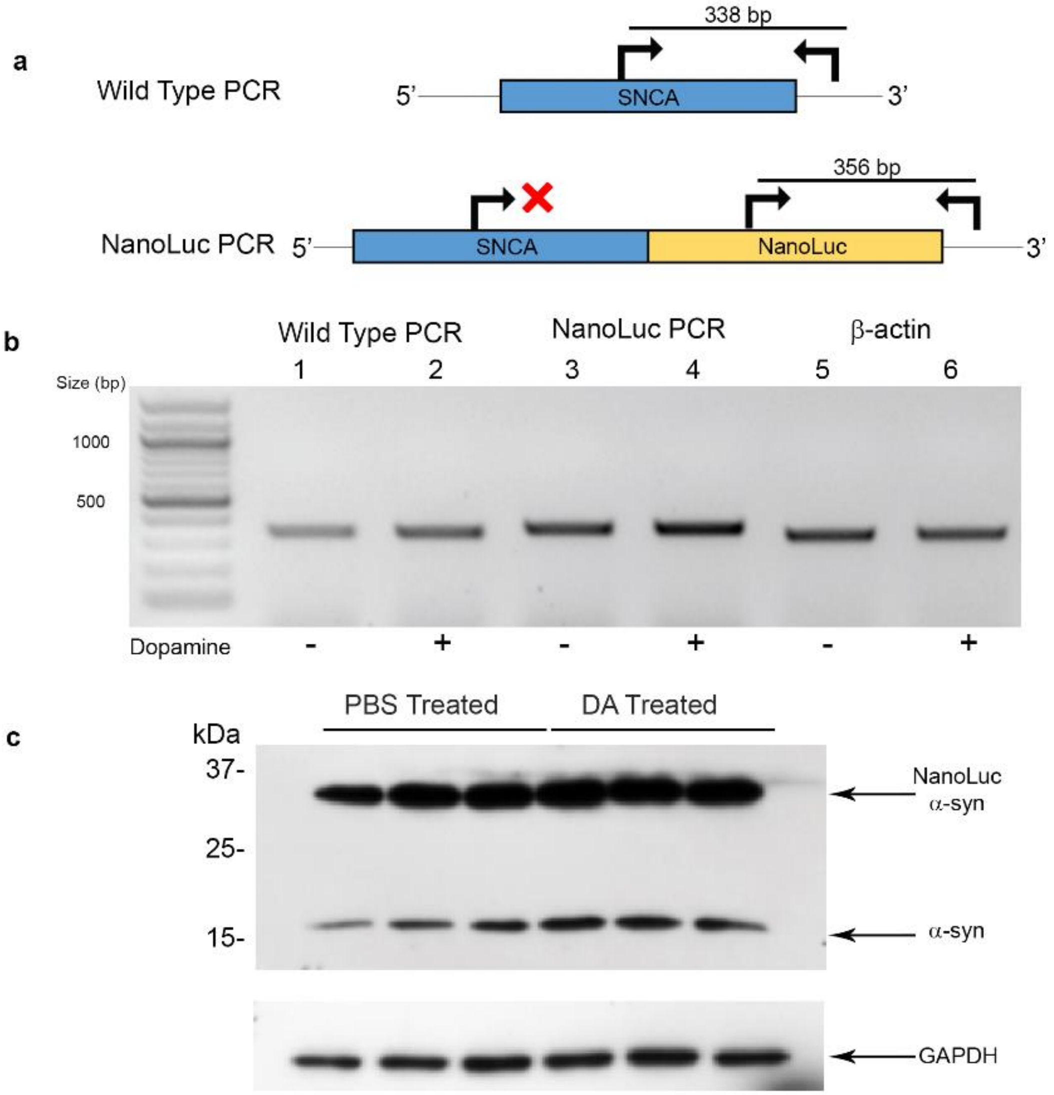

Supplement: Supplementary Information [file srep45883-s1.pdf]
